# Idiopathic pulmonary fibrosis telemedicine management during COVID-19 outbreak

**DOI:** 10.1515/med-2022-0466

**Published:** 2022-04-07

**Authors:** Anna Agnese Stanziola, Andrea Salzano, Rossella D’Angelo, Alberto Maria Marra, Lorena Gallotti, Roberta D’Assante, Danilo Pentangelo, Brigida Ranieri, Eduardo Bossone, Antonio Cittadini

**Affiliations:** Section of Respiratory Disease, Department of Clinical Medicine and Surgery, Monaldi Hospital, Federico II University, 80131 Naples, Italy; IRCCS Synlab SDN, Diagnostic and Nuclear Research Institute, 80143, Naples, Italy; Department of Translational Medical Sciences, Federico II University, Naples, Italy; Department of Cardiology, AORN A Cardarelli, Naples, Italy

**Keywords:** Telemedicine, idiopathic pulmonary fibrosis, COVID-19, outcomes, SARS-CoV-2, prognosis

## Abstract

The present report investigates the impact of a Telemedicine Service (TMS) on the management of Idiopathic Pulmonary Fibrosis (IPF) during coronavirus disease of 2019 (COVID-19) outbreak in Italy. The TMS comprised 3 phone numbers, active 12 h per day, and an email address, monitored every 4 h by trained physicians; chat- and videoconference-services were also offered. At the end of the study period, our staff contacted all patients, to get information about the final outcome (i.e. composite hospitalisations/all causes of death). Outcomes were compared with a cohort of patients who attended our unit in the same period of the previous year (when no TMS was available). 189 patients participated in the present study. From 11th March to 4th May 2020, 61% of patients made at least one TMS access, mostly by emails (53%), followed by phone calls (33%). With regard to the primary outcome, TMS patients experienced a significant lower rate of events of the 182 patients of the no-TMS cohort (*p* < 0.001). Specifically, a significant difference was observed for IPF hospitalisation (*p* < 0.001) whereas no differences were observed with regard to deaths (*p* = 0.64). TMS permits patients to be followed up even during COVID-19 lockdown, with an encouraging impact on outcomes.

## 1 Introduction

A few weeks after the first case of person-to-person transmission of severe acute respiratory syndrome coronavirus 2 (Sars-CoV-2) was confirmed in a small town in the Northern Italy [1], a governmental Decree-Law imposed a global lockdown in Italy, with the aim of reducing the spread of coronavirus disease of 2019 (COVID-19). As a result, national healthcare system has been affected by several restrictions, with outpatient clinics and day-services suspended and inward access allowed only for urgent events. Despite leading to a reduction in the risk of in-hospital COVID-19 spread, as a downside, a huge decrease in the clinic assistance of chronic diseases – e.g. Idiopathic Pulmonary Fibrosis (IPF) – was expected. Therefore, our IPF university tertiary referral centre [2,3] set up a Telemedicine Service (TMS) to guarantee clinical assistance to patients affected by this chronic disease during the COVID-19 pandemic.

Aims of the present report were to investigate the impact of TMS on the management of IPF during COVID-19 outbreak, and to compare outcomes (i.e. composite of hospitalisation/death) with the identical period (11th March–4th May) of the previous year, when TMS was not available.

## 2 Materials and methods

Our TMS comprised 3 phone numbers, active 12 h per day, and an email address, monitored every 4 h by trained physicians; chat- and videoconference-services were also offered, using most popular smartphone applications. On purpose, the TMS was based mostly on phone calls, to avoid possible social disparities (e.g. accessibility of service only to people with available technologies and/or capacities to use the service) [4]; indeed, as minimum hardware requirements, patients were asked to have free access to a phone, available at their need. All accesses were on voluntary basis by the patients, who were encouraged to use the TMS for all clinical needs. General measures to prevent COVID-19 diffusion (e.g. frequent handwashing, use of face masks in public places, social distancing, and self-isolating) were prescribed. Patients were encouraged to assess parameters such as blood pressure and oxygen saturation (SpO_2_) at least three times per week. With regard to IPF specific treatment, we recommended the prosecution of all drugs as per medical standard [5]. At the end of the study period, our staff contacted all patients, to get information about the final outcome.

Data are expressed as mean value ± SD or median (interquartile range [IQR]) wherever appropriate. Statistical analysis was performed using SPSS version 26 (IBM, New York, NY, USA) running on a MAC OSX 10.15.5 (Apple, Cupertino, CA, USA). Pearson’s chi-squared and Fisher’s exact tests were used to compare the primary outcomes (defined as a composite of hospitalisation/death, all-cause hospitalisations, or all-cause mortality). A *p* value <0.05 was considered significant.

The need for the patient’s informed consent was waived due to the observational design of the study, and because patients’ information have been anonymised before data analysis.

## 3 Results

**Figure 1:**
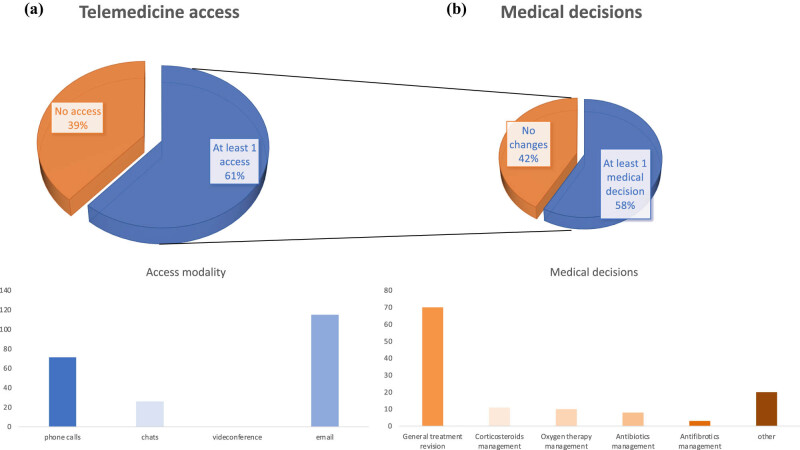
(a) Patients who accessed TMS and access modality. (b) Outcome of the accesses performed and medical decisions.

One hundred and eighty-nine patients participated in the present study; outcomes were compared with data from 182 IPF patients who attended our unit in the same period of 2019 (Table 1). From 11th March to 4th May 2020, 61% of patients made at least one TMS access, mostly by emails (53%), followed by phone calls (33%). Overall, 56% of contacts led to a clinical decision (adjustment of corticosteroids/antibiotics/antifibrotic doses, oxygen management, and others), based on clinical data reported by patients during the contact (e.g. blood pressure and SpO_2_) (Figure 1). No differences were found with regard to education and social status of patients who accessed the TMS compared to patients who did not (data not shown). With regard to the primary outcome, nine patients experienced the primary endpoint. Specifically, four patients deceased (all of them were at advanced stage of IPF before the lockdown), whereas five patients were hospitalised (one for stroke, two for exacerbation of the IPF, and one for non IPF-related surgery); notably, only 1 of our IPF patients got COVID-19, with an unexpected positive outcome after hospitalisation. Pearson’s chi-squared and Fisher’s exact tests showed that patients in 2020 cohort (when TMS was available) were less likely to experience the primary outcome (*X* ^2^ [degree of freedom 1, cases = 371] 21.8, *p* < 0.001) than the 2019 cohort (i.e. TMS not offered). Specifically, a significant difference was observed for IPF hospitalisation (*p* < 0.001), whereas no differences were observed with regard to deaths (*p* = 0.64).

**Table 1:** Demographic characteristics at baseline, telemedicine data, and outcomes

Variables	Cohort 2020 (*n* = 189)	Cohort 2019 (*n* = 182)	*p*
**Demographics**			
Age (year)	71.8 ± 7.4	70 ± 8.1	ns
Sex (male/female)	114/80	115/64	ns
Systolic blood pressure (mm Hg)	126 ± 5.7	125 ± 4.3	ns
Diastolic blood pressure (mm Hg)	80.2 ± 7.4	79.3 ± 6.7	ns
Heart rate (bpm)	78.9 ± 8.6	78.3 ± 7.3	
BMI (kg/m^2^)	31.9 ± 6.3	29.7 ± 5.2	ns
Smokers (%)	59 (31)	60 (33)	ns
FVC (% predicted)	75.9 ± 23.3	76.5 ± 22.4	ns
SpO_2_ (%)	91.28 ± 5.21	90.71 ± 4.87	ns
**Medication**			
O_2_ therapy (%)	52 (27)	51 (28)	ns
Corticosteroids (%)	60 (32)	58 (31)	ns
Antifibrotics (pirfenidone/nintedanib/none)	79/105/5	72/107/3	ns
**Telemedicine**			
Total number of accesses	215	—	—
Patients with at least 1 access (%)	116 (61%)	—	—
**Type of access**			
Phone calls (%)	71 (33)	—	—
Chat (%)	26 (12%)	—	—
Video (%)	0 (%)	—	—
Emails (%)	115 (53%)	—	—
Patients needing at least one clinical intervention	110 (58%)	—	—
Number of clinical interventions	122 (56%)	—	—
**Type of clinical intervention**			
Monitoring for treatment confirmation	70	—	—
Corticosteroids management	11	—	—
Oxygen therapy management	10	—	—
Antibiotics management	8	—	—
Antifibrotics management	3	—	—
Others^#^	20	—	—
**Outcome**			
	2020 lockdown	Same period in 2019	*p*
Composite hospitalisation/death	9	38	0.001*
Hospitalisations	5	33	0.001*
Deaths	4	5	ns

BMI: body mass index; FVC: forced vital capacity; SpO_2_: blood oxygen saturation level. Period of observation: 11th March–4th May 2020 for COVID-19 lockdown cohort, and 11th March–4th May 2019 for control cohort; * *p* < 0.01; ^#^ other interventions: diuretics prescription, arterial hypertension management, blood analyses monitoring, general advice. Data expressed as mean value ± standard deviation or percentages.

## 4 Discussion

The present investigation represents the first study designed to investigate the utility of telemedicine in the management of IPF during the lockdown due to Covid-19 outbreak. Telemedicine represents one of the most promising methods to improve quality and healthcare availability during period of limited direct contact between patients and healthcare professionals. Using worldwide available technologies (analogic phones, emails, smartphones, apps), our large cohort of IPF patients has been followed up even during the COVID-19 outbreak, with a positive impact on the general IPF management, as shown in other diseases (e.g. heart failure) [6]. About half of the patients needed at least one TMS access, and about 50% of these accesses led to a clinical decision, confirming the usefulness of our TMS. Intriguingly, even if our TMS was based mostly on phone calls to avoid possible social inequality, in our cohort, the most frequent way to access was by email; this was the opposite of the findings we observed when a similar TMS has been applied in another chronic disease (i.e. heart failure), with patients preferring phone calls [6]. A possible explanation is the different nature of the patients’ request between these two cohorts (confirmation of treatment vs changes in symptoms relief drugs dosage, IPF vs heart failure cohort), leading to the hypothesis that the urgency of the need was the most impacting factor on the means of access to the TMS. A recent randomised controlled trial showed that an IPF home monitoring program did not improve the quality of life but psychological well-being, with great appreciation by patients [7]; in this regard, our patients expressed a great appreciation about our TMS. Further, home monitoring program allowed individually tailored medication adjustments and early detection of intercurrent events [7], in line with the results of the present report and with a similar experience in a different chronic disease [6]. In addition, our TMS allowed to anticipate hospitalisation of the patient affected only by COVID-19 in our cohort, who was referred to the closer COVID-19 hospital after a TMS access. Altogether, these findings further support the idea that a TMS can help in addressing the challenges that physical isolation is creating in patients with chronic diseases during the COVID-19 outbreak [8,9]. Notably, recently the development of telemedicine has been pointed out as one of the key priorities in the agenda for COVID-19 research in respiratory disease, and being an instrument able to improve patient management, allowing effective isolation and disease monitoring [10]. Finally, the use of telephone or video appointments has been described as a useful strategy to reduce potential exposure to COVID-19 for patients with fibrotic interstitial lung disease [11].

### 4.1 Study limitations

As study limitations, our report shows data from a single centre experience. Further, some variables (e.g. age of patients, availability of modern technologies, and socio-cultural barriers) could impact the capacity of the patients to access the TMS service, representing a limitation of TMS method itself. However, we included a direct phone number in our TMS, available to all patients and their caregivers, trying to minimise this possible bias. Further, the team provided support and training to the caregivers when patients were not able to access TMS by their own.

## 5 Conclusion

In conclusion, our TMS permits our large cohort of IPF patients to be followed up even during COVID-19 lockdown, with an encouraging impact on IPF outcome. With the aim of reducing possible socio-cultural limits, it is important that TMS comprises simple phone based service too, and not only high-technologies methods.

The present report confirms telemedicine as a valuable tool in IPF management and shows its feasibility in IPF management during COVID-19 outbreak.
